# Portuguese Version of the HLS-EU-Q6 and HLS-EU-Q16 Questionnaire: Psychometric Properties

**DOI:** 10.3390/ijerph20042892

**Published:** 2023-02-07

**Authors:** Ana Rita Pedro, Beatriz Raposo, Luís Luís, Odete Amaral, Ana Escoval, Sara Simões Dias

**Affiliations:** 1 NOVA National School of Public Health, Comprehensive Health Research Center (CHRC), NOVA University Lisbon, 1600-560 Lisboa, Portugal; 2 NOVA National School of Public Health, Public Health Research Center, CISP, NOVA University Lisbon, 1600-560 Lisboa, Portugal; 3 ciTechCare—Center for Innovative Care and Health Technology, Campus 5, Polytechnic of Leiria, 2410-541 Leiria, Portugal; 4 School of Health Sciences, Campus 2—Morro do Lena, Polytechnic of Leiria, 2411-901 Leiria, Portugal; 5 UICISA: E Research Centre, Public Health, Family and Community Nursing Unit, Health School of Viseu, Polytechnic Institute of Viseu, 3500-843 Viseu, Portugal

**Keywords:** questionnaire, psychometrics, HLS-EU-PT-Q16, HLS-EU-PT-Q6, HLS-EU-PT, health literacy

## Abstract

Health literacy refers to the competencies of individuals and the general population to navigate all the areas of health care, making health decisions. Health professionals need a set of skills and information to adapt to people’s health literacy. To succeed, it is crucial to determine the health literacy level of a population, in this case, the Portuguese. This study aims to measure the psychometric properties of the Portuguese version of HLS-EU-Q16 and HLS-EU-Q6 from the long form of HLS-EU-Q47, already validated for Portugal. To analyse these results, a comparison was made with the HLS-EU-PT index. Spearman correlation analysis was performed between the single items and scale scores. Cronbach’s alphas for all the indexes were calculated. For the statistical analysis, SPSS (version 28.0) was used. Cronbach’s alpha coefficient for HLS-EU-PT-Q16 internal consistency was 0.89 overall, and for HLS-EU-PT-Q6 was 0.78 overall. Indexes were not normally distributed, and the Spearman correlation was computed. The correlation between G HL47 and G HL16 indexes was ρ = 0.95 (*p* < 0.001), and between G HL6 and HLS-EU-PT-Q6 was perfect. The HLS-EU-PT-Q16 and HLS-EU-PT-Q6 are concise and present adequate psychometric properties to measure the HL level of the Portuguese population. However, more similarities are found between the 47-item and the 16-item forms.

## 1. Introduction

Over the years, many definitions and conceptual frameworks of health literacy arose; however, in 2012, Sørensen, K. et al., conducted a systematic review that synthesised and captured the essence of all of them and stated that: “Health literacy is linked to literacy and entails people’s knowledge, motivation and competences to access, understand, appraise and apply health information in order to make judgments and take decisions in everyday life concerning healthcare, disease prevention and health promotion to maintain or improve quality of life during the life course.” [1]. For the authors, health literacy contributes to the improvement of knowledge and instruction of people in the three health domains: healthcare, disease prevention and health promotion [1].

Throughout the years, several instruments have been developed to determine the health literacy level of a population. However, none of them fully covered all the concepts and areas identified by health literacy, instead focusing only on some specific aspects of it [2,3]. To properly evaluate the population health literacy level, we need an instrument that is able to cover all of its domains and aspects [1]. The ideal tool should not only be able to determine health literacy level of the overall population, but also identify groups behind the different levels, allowing the creation of adequate strategies and intervention to approach each group [3]. Due to the existence of this unmet need, the HSL-EU consortium, which happened between 2009 and 2012, developed a tool to measure health literacy in populations on the basis of the definition and conceptual model proposed by Sørensen, K. et al. The European Health Literacy Survey Questionnaire (HLS-EU-Q) was developed for measuring the health literacy of the population in general and in this way get a broad public health perspective. The 47-item questionnaire measures health literacy through four information processing competences—accessing, understanding, appraising and applying to make decisions related to health information, and three domains—health care, disease prevention and health promotion; all of which assesses the 12 subdomains of health literacy [2,4].

In 2014, Portugal translated, validated and applied the survey within the population (HLS-EU-PT). The responses indicated that 61% of the sample population surveyed had a problematic or inadequate level of general health literacy. This is higher than the average of the nine other countries previously surveyed (49.2%). Due to the time and cost of administering the HLS-EU-PT, there was a proposal for a new study in Portugal to validate the short form of the same instrument, which has 16 items [5].

The current study is the result of that suggestion of the Portuguese validated study (47-item). Its objective is to measure the psychometric properties of the Portuguese version of the HLS-EU-PT-Q16 and HLS-EU-PT-Q6, the short and short-short forms of the 47-items version questionnaire (HLS-EU-Q47). Several countries have already translated and validated one of these instruments (short forms), or have proposed to do so [6,7,8,9,10,11,12,13,14].

## 2. Materials and Methods

### 2.1. Aim

This study aims to measure the psychometric properties of the Portuguese versions of the HLS-EU-Q6 and HLS-EU-Q16 questionnaires.

### 2.2. Design

In order to assess the psychometric properties (internal consistency and Spearman correlations), a methodological analysis of the HLS-EU-PT-Q16 and HLS-EU-PT-Q6 instrument was conducted by using the data already collected in the study that validated the Portuguese version (HLS-EU-PT) [5].

### 2.3. Characteristics of Participants

The data of HLS-EU-PT were collected in various public spaces and in different periods of the day. To respect the fair geographical distribution and considering that the Portuguese population was of 10,626,008 individuals, the sample was calculated according to the population density of the territorial units for statistics (NUTS) (specifically the NUTS II regions), and was stratified by gender. The dataset contained 1004 people, with age ≥ 16 years old; 60.4% of them were female. Almost half of the people were single (49.6%). The most common academic qualification was high school (36.3%), followed by undergraduate degree (32.7%). Finally, 40.7% had a full-time job and 30.2% were students [4,5].

### 2.4. Adaptation for Portuguese Language

The HLS-EU-PT instrument with 47 items had already been translated to the Portuguese language [5]. The translation method used was the Hill and Hill translation–retroversion [5,15], in order to maintain semantic and cultural equivalence between the original and the Portuguese version. The translation was followed by a pre-test in a sample with 250 Portuguese individuals aged ≥16 years old [5].

### 2.5. Characterization of Instrument—HLS-EU-PT-Q16

The HLS-EU-PT-Q16 questionnaire is a short-form and is composed of 16 items selected from the larger form of 47 items. It covers the three domains: healthcare, disease prevention, and health promotion. To correctly evaluate the health literacy of the questionees, they must have answered at least 14 of the 16 questions. The 16 items were then dichotomized: “difficult” and “very difficult” answers were given the value of 0, and “easy” and “very easy” answers were given the value of 1. The sum of the values attributed to each of the 16 items is the level of health literacy of each individual. A total sum of ≥13 is considered to be an “adequate” level of health literacy, a value between 9 and 12 is considered to be a “problematic” level of health literacy, and any value ≤8 characterizes individuals with an “inadequate” level of health literacy [8,9,10,16,17]. Moreover, in order to ensure an effective comparison between the subdomains, the indices were standardized on the general HL index, a variable metric scale between 0–50. The general HL index (G-HL16 index) was calculated as follows: G-HL16 index = (mean − 1) × (50/3). All participating items for everyone was considerate as the “mean”. Four levels of HL were defined: inadequate (0–25), problematic (25.1–33), sufficient (33.1–42), and excellent (42.1–50) [10].

### 2.6. Characterization of Instrument HLS-EU-PT-Q6

The HLS-EU-PT-Q6 questionnaire is composed of 6 items selected from the larger HLS-EU-PT. It also covers the same three sub-domains and uses the same Likert scale as HLS-EU-PT-Q16. The scale used was the Likert scale, composed of options that range from “very easy” to “very difficult”, and “don’t know” answers are coded as missing values [18]. Only respondents who answered at least 5 items were considered. The scores were considered as follows: HLS-EU-PT-Q6 using the mean of all participants answers that varies from 1 to 4 (1–2, inadequate, 2.1–3 problematic and 3.1–4 adequate) [18], and the G-HL6 index that was calculated as G-HL6 index = (mean − 1) × (50/3); the same four levels of HL were considered.

### 2.7. Statistical Analysis

Data are presented as percentages and as means ± standard deviations. For all indexes, normality was calculated. To analyse the results obtained, a comparison was made with the HLS-EU-PT index. Spearman correlation analysis was performed between the single items and scale scores. Cronbach’s alphas for all the indexes were calculated as a measure of internal consistency. The software used for the statistical analysis was SPSS (version 28.0, IBM Corp., Armonk, NY, USA).

## 3. Results

The sample consists of 977 individuals.

### 3.1. Responses to the HLS-EU-PT-Q16 and HLS-EU-PT6 Items

Table 1 reports the responses to the HLS-EU-Q items. Three participants and one were excluded from generating the score of HLS-EU-PT-Q16 and HLS-EU-PT-Q6, respectively, due to an excess of “don’t know/ refusal” answers. The percentages of “don’t know/refusal” responses varied from 0% to 1.2%.

Items 12, 11, 8 and 5 reported a highest percentage of “very difficult” responses (5.2%, 4.8% and 4.2%). Items 10, 4 and 9 had the highest percentages of “very easy” responses (39.2%, 36.8% and 36.3%, respectively).

### 3.2. Spearman Correlation Analysis

All the 16 items are significantly correlated, with correlations varying between 0.183 and 0.547 (Table 2).

Indexes were not normally distributed, and the Spearman correlation was computed. HLS-EU-PT, HLS-EU-PT-Q16, HLS-EU-PT-Q6, G-HL16 and G-HL6 indexes were strongly correlated (Table 3). Further, we highlight the results obtained in the correlation between G HL47 and G HL16 indexes (ρ = 0.949, *p* < 0.001), and G HL6 and HLS-EU-PT-Q6 were a perfect correlation.

### 3.3. Cronbach’s Alpha Coefficient

Cronbach’s alpha coefficient for HLS-EU-PT-Q16 internal consistency were as follows: 0.783 for the health care subdomain, 0.724 for the disease prevention subdomain, 0.703 for the health promotion subdomain, and 0.89 for HLS-EU-PT-Q16 overall. Regarding HLS-EU-PT-Q6, the Cronbach’s alpha coefficient obtained was 0.777 overall.

### 3.4. Level of Health Literacy

Considering each of the indices in its calculation formula, the results are as follows. For the formula, HL index = (mean − 1) × (50/3). It is worth mentioning the evident similarity between G HL47 and G HL 16 in the levels of health literacy (Table 4).

For the formula using the sum of the scores of each item that varied between 0 and 1, the results are present in Table 5.

Comparing the results of Table 4 with Table 5, we may assume that HLS-EU-PT-Q16 is similar to G HL16. The values for the inadequate and problematic HL level are similar, and the adequate HL level value has an approximate value of the sum of sufficient and excellent HL levels.

## 4. Discussion

Health information must meet the patient’s needs. To adapt the information to these needs, it is necessary that a health literacy instrument be able to assess the areas that need to be addressed, as well as the person’s capabilities [3].

In this rationale, and as we have seen in the results of this study, we analysed from the short forms (HLS-EU-PT-Q16 and HLS-EU-PT-Q6) that people report with a higher percentage of “very difficult” the items: 12—“decide how you can protect yourself from illness based on information in the media?”; 11—“judge if information about health risks in the media is reliable?”; 8—“find information on how to manage mental health problems like stress or depression?”; and 5—“ judge when you may need to get a second opinion from another doctor?”, with 5.2%, 4.8%, 4.2 and 4.2%, respectively. When comparing these results with the long form validated for Portuguese, the results are similar. Item 12 corresponds to item 31 of the 47-item form, item 11 corresponds to item 28 of the 47-item form, item 8 corresponds to item 18, and, finally, item 5 corresponds to item 11 of the HLS-EU-PT. In the validity version for Portuguese (HLS-EU-PT), the percentages of answers in the “very difficult” are 5.4%, 4.8%, 4.2% and 4.2%, respectively [4].

To understand the dimensions represented here, items 8, 11 and 12 are within the “disease prevention” domain, and item 5 is within the “health care” domain. Another aspect to be taken into account are items 11, 8 and 5 in the short-short form of 6 items.

However, people report with a higher percentage of “very easy” item 4 from the “health care” domain, and items 9 and 10 from the “disease prevention” domain. Item 10 (“understand why you need health screenings?”) presents the highest percentage with 39.2% of answers; item 4 (“understand your doctor’s or pharmacist’s instruction on how to take a prescribed medicine”) presents the second highest percentage with 36.8%, and item 9 (“understand health warnings about behaviour such as smoking, low physical activity and drinking too much?”) presents 36.3%. When comparing these results with the 47-item form validated for Portuguese [4], we understand that the results are also similar. Item 4 corresponds to item 8 of the 47-item form containing 36.8% of the answers in the “very easy”; item 9 corresponds to item 21 of the 47-item form, with 36.7% of people reporting “very easy”, and item 10 corresponds to item 23 of the 47-item form with 39.7% of the answers in the “very easy” [4].

In this study, it can be observed that the HLS-EU-PT, HLS-EU-PT-Q16, HLS-EU-PT-Q6, G-HL16 and G-HL6 indexes were strongly correlated, with a correlation above 0.8. This highlighted that the results obtained in the correlation G HL47 and G HL16 indexes are ρ = 0.949, with *p*-value < 0.001, and the correlation between G HL6 and HLS-EU-PT-Q6 ρ = 1, *p*-value < 0.001.

Reliability is the ability of the instrument to reproduce a result consistently. This is related, above all, with the stability, internal consistency and equivalence of a measure; this is not a fixed property of an instrument, and can be influenced by some factors (e.g., the function of the instrument, population, context, etc.) [19,20].

Internal consistency is an important measurement property for questionnaires that intend to measure a single construct using multiple items. Cronbach’s alpha is frequently used to measure the internal consistency [19,21], although it is important to take into account that the alpha values are influenced by the number of items [19,22,23].

“A low internal consistency could mean that the items measure different attributes or the subjects’ answers are inconsistent” [20].

A questionnaire’s scale with more than 14 items has a good internal consistency if the Cronbach´s alpha is greater than 0.7 [22]. If alpha = 1, then the items are perfectly related with one another; if, on the contrary, alpha = 0, the items are not related between them [21]. In our study, the Cronbach´s alpha coefficient for the HLS-EU-PT-Q16, for the domains, ranged from: 0.783 for health care; 0.724 for disease prevention, and 0.703 for the health promotion. The total value obtained was 0.89. With these results, it was verified that the HLS-EU-PT-Q16 has internal consistency. Regarding HLS-EU-PT-Q6, the Cronbach’s alpha coefficient obtained was 0.777 overall. Pelikan et al. reported a Cronbach’s alpha on the HLS-EU-Q6 of 0.803 [18]. A study measuring health literacy in Italy and validating the HLS-EU-Q16 and the HLS-EU-Q6 for the Italian language obtained Cronbach’s alphas of 0.799 and 0.672, respectively [10]. Values closer to 0.60 are satisfactory [19].

Considering the literacy level presented in Table 4 of the results, calculated by the form: HL index = (mean − 1) × (50/3), it presents some differences between the percentages of HL levels between the 47-, 16- and 6-item forms. This phenomenon is also presented in Pelikan et al. [18]. However, in this study, the forms that show the smallest difference between the percentages are the 47-item and 16-item.

### Limitation

This study presents some limitations. Data analysis was performed using the database of HLS-EU-PT, already applied in Portugal, and the original structure of HLS-EU-Q16 and HLS-EU-Q6 does not allow the selection of the items according to the data. It should also be noted that confirmatory factor analysis (CFA) can only be applied in a further study. Another limitation that should be considered is the fact that the “Apply” level of information processing is not represented in the sub-dimension of Health Promotion in HLS-EU-PT-Q16, and, in the HLS-EU-PT-Q6, “Access” and “Understand” in the sub-dimension of Health Care, “Understand” and “Apply” in Disease Prevention, and “Appraise” and “Apply” in the Health Promotion sub-dimension are not represented.

## 5. Conclusions

The short versions of HLS-EU-PT—(HLS-EU-PT-Q16 and HLS-EU-PT-Q6)—are concise and present adequate psychometric properties to measure the health literacy level of the Portuguese population. However, the forms that show more similarities are the 47-item and the 16-item forms of the HLS-EU-PT.

## Figures and Tables

**Table 1:** Responses to the HLS-EU-PT-Q16 /6 items (n; percentages).

Area	On a Scale from Very Easy to Very Difficult, How Easy Would You Say It Is To	Very Easy	Easy	Difficult	Very Difficult	Don’t Know/Refusal
HC	1. find information on treatments of illness that concern you	144; 14.8%	564; 58.0%	226; 23.2%	33; 3.4%	6; 0.6%
HC	2. find out where to get professional help when you are ill?	176; 18.1%	515; 52.9%	252; 25.9%	26; 2.7%	4; 0.4%
HC	3. understand what your doctor says to you?	207; 21.3%	589; 60.5%	166; 17.1%	8; 0.8%	3; 0.3%
HC	4. understand your doctor’s or pharmacist’s instruction on how to take a prescribed medicine	358; 36.8%	517; 53.1%	81; 8.3%	11; 1.1%	6; 0.6%
HC	5. judge when you may need to get a second opinion from another doctor? ^†^	93; 9.6%	479; 49.2%	352; 36.2%	41; 4.2%	8; 0.8%
HC	6. use information the doctor gives you to make decisions about your illness? ^†^	98; 10.1%	588; 60.4%	250; 25.7%	31; 3.2%	6; 0.6%
HC	7. follow instructions from your doctor or pharmacist?	345; 35.5%	541; 55.6%	72; 7.4%	11; 1.1%	4; 0.4%
DP	8. find information on how to manage mental health problems like stress or depression? ^†^	133; 13.7%	456; 46.9%	334; 34.3%	41; 4.2%	9; 0.9%
DP	9. understand health warnings about behaviour such as smoking, low physical activity and drinking too much?	353; 36.3%	529; 54.4%	81; 8.3%	10; 1.0%	0; 0.0%
DP	10. understand why you need health screenings?	381; 39.2%	504; 51.8%	69; 7.1%	7; 0.7%	12; 1.2%
DP	11. judge if the information on health risks in the media is reliable? ^†^	94; 9.7%	410; 42.1	410; 42.1%	47; 4.8%	12; 1.2%
DP	12. decide how you can protect yourself from illness based on information in the media?	91; 9.4%	448; 46.0%	371; 38.1%	51; 5.2%	12; 1.2%
HP	13. find out about activities that are good for your mental well-being? ^†^	236; 24.3%	512; 52.6%	192; 19.7%	33; 3.4%	0; 0.0%
HP	14. understand advice on health from family members or friends?	133; 13.7%	595; 61.2%	205; 21.1%	33; 3.4%	7; 0.7%
HP	15. understand information in the media on how to get healthier? ^†^	185; 19.0%	584; 60.0%	184; 18.9%	18; 1.8%	2; 0.2%
HP	16. judge which everyday behaviour is related to your health?	206; 21.2%	585; 60.1%	150; 15.4%	23; 2.4%	9; 0.9%

Legend—HC: health care; DP: disease prevention; HP: health promotion; ^†^ Items included in the HLS-EU-Q6.

**Table 2:** Spearman correlation analysis of the HLS-EU-PT-Q16 /6 items.

	Item 1	Item 2	Item 3	Item 4	Item 5 ^†^	Item 6 ^†^	Item 7	Item 8^†^	Item 9	Item 10	Item 11 ^†^	Item 12	Item 13 ^†^	Item 14	Item 15 ^†^	Item 16
Item 1	1															
Item 2	0.367 *	1														
Item 3	0.354 *	0.353 *	1													
Item 4	0.273 *	0.321 *	0.493 *	1												
Item 5 ^†^	0.313 *	0.338 *	0.318 *	0.299 *	1											
Item 6 ^†^	0.298 *	0.355 *	0.411 *	0.333 *	0.389 *	1										
Item 7	0.241 *	0.320 *	0.362 *	0.538 *	0.249 *	0.309 *	1									
Item 8 ^†^	0.412 *	0.347 *	0.266 *	0.267 *	0.306 *	0.355 *	0.270 *	1								
Item 9	0.267 *	0.341 *	0.333 *	0.480 *	0.283 *	0.297 *	0.495 *	0.328 *	1							
Item 10	0.317 *	0.360 *	0.367 *	0.450 *	0.271 *	0.273 *	0.496 *	0.296 *	0.547 *	1						
Item 11^†^	0.296 *	0.307 *	0.220 *	0.183 *	0.397 *	0.371 *	0.184 *	0.402 *	0.204 *	0.229 *	1					
Item 12	0.328 *	0.297 *	0.223 *	0.193 *	0.323 *	0.370 *	0.183 *	0.365 *	0.219 *	0.226 *	0.531 *	1				
Item 13 ^†^	0.299 *	0.319 *	0.261 *	0.303 *	0.297 *	0.305 *	0.294 *	0.417 *	0.405 *	0.356 *	0.385 *	0.339 *	1			
Item 14	0.204 *	0.278 *	0.232 *	0.279 *	0.255 *	0.304 *	0.249 *	0.266 *	0.276 *	0.263 *	0.307 *	0.367 *	0.304 *	1		
Item 15 ^†^	0.242 *	0.314 *	0.293 *	0.418 *	0.306 *	0.328 *	0.422 *	0.298 *	0.456 *	0.374 *	0.399 *	0.354 *	0.407 *	0.415 *	1	
Item 16	0.250 *	0.316 *	0.319 *	0.356 *	0.369 *	0.307 *	0.386 *	0.324 *	0.423 *	0.402 *	0.340 *	0.299 *	0.408 *	0.291 *	0.439 *	1

Legend—^†^ items included in the HLS-EU-PT-Q6; * *p*-value < 0.001.

**Table 3:** Spearman correlation analysis of the HLS-EU-PT, HLS-EU-PT-Q16, HLS-EU-PT-Q6, G-HL16 and G-HL6 indexes.

	G HL 47	G HL 16	G HL 6	HLS-EU-PT-Q16	HLS-EU-PT-Q6
G HL 47	1				
G HL 16	0.949 *	1			
G HL 6	0.874 *	0.896 *	1		
HLS-EU-PT-Q16	0.852 *	0.876 *	0.866 *	1	
HLS-EU-PT-Q6	0.873 *	0.896 *	1 *	0.871 *	1

Legend—* *p*-value < 0.001.

**Table 4:** Level of health literacy (HL index).

	G HL 47	G HL 16	G HL 6
Inadequate	163 (17.0%)	153 (15.7%)	338 (34.7%)
Problematic	425 (44.4%)	379 (39.0%)	263 (27.0%)
Sufficient	289 (30.2%)	355 (36.5%)	303 (31.1%)
Excellent	80 (8.4%)	86 (8.8%)	69 (7.1%)

**Table 5:** Level of health literacy.

	HLS-EU-PT-Q16	HLS-EU-PT-Q6
Inadequate	156 (16.0%)	65 (6.9%)
Problematic	323 (33.2%)	672 (71.8%)
Adequate	495 (50.8%)	199 (21.3%)

## Data Availability

Data can be found at the NOVA National School of Public Health and can be accessed by contacting corresponding author.
